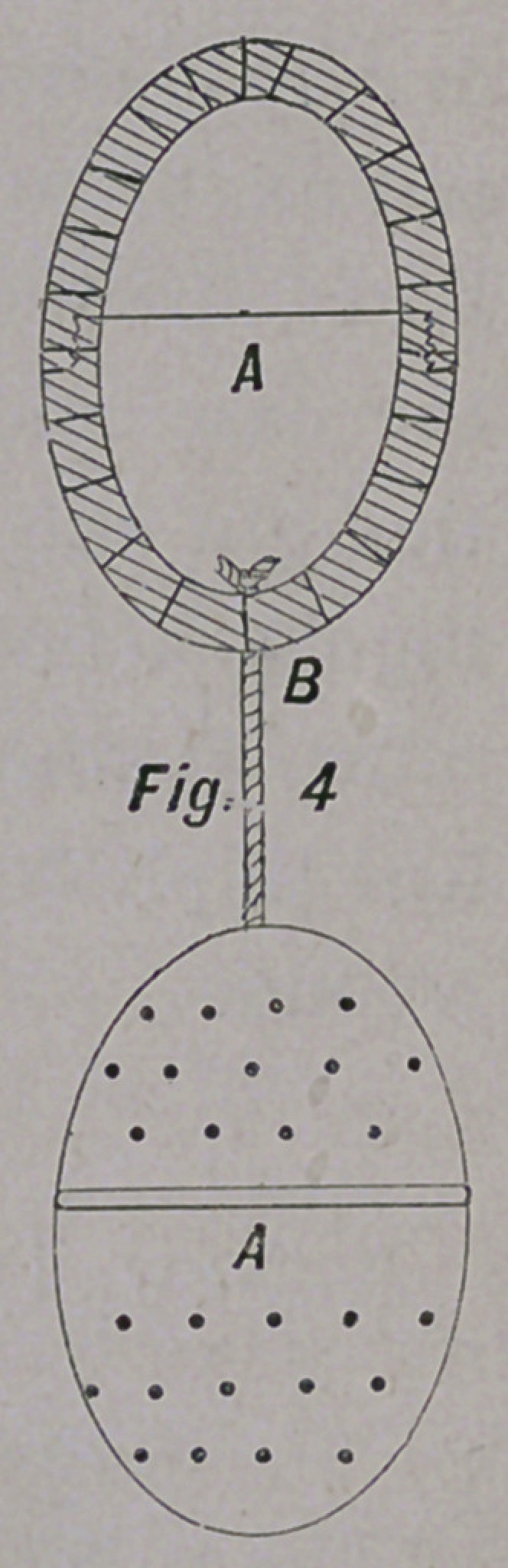# A New Treatment for Hemorrhoids—A Pile Pessary

**Published:** 1882-12

**Authors:** Thomas Lothrop


### A NEW TREATMENT FOR HEMORRHOIDS—A PILE PESSARY.

REPORTED BY THOMAS LOTHROP, M. D.

The sufferings attending a severe case of piles have excited the ingenuity of both the surgeon and the patient. The abhorrence of the knife or the ligature has driven many into the hands of quacks, or to the employment of nostrums, with the almost invariable result of failure and consequent despair.

The device which it is the object of this paper to bring before the profession was the outcome of an attack of this malady in the person of a prominent merchant of this city, who, after suffering for many months, and being almost incapacitated from business, resorted to the use of these pessaries with the result of almost complete relief. The principle upon which the pessaries acts is that of pressure upon the distended hemorrhoidal veins and the support given to the relaxed rectal walls. This same principle is utilized in the treatment of uterine displacements, and, when wisely directed, is productive of good results. Its application to the diseases of the rectum is certainly philosophical, and, judging from its success in the case above referred to, is worthy of the attention of the profession.

The accompanying cuts will give the reader an idea of the form and size of the pessary here recommended. They are olive-shaped, and can be made of wood, celluloid, ivory, or hard rubber.

No. 1 *A* is solid; No. 2 *A* is solid, and corrugated on its surface; No. 3 *A* is hollow, and smooth on its surface; No. 4 is hollow and perforated. The pessaries are united by a silk thread, *B*.

The directions for the use and application of these pessaries are as follows: After the bowels have been moved, the patient inserts the first pessary, and the second ball follows immediately. The hemorrhoids are thus pushed up in their proper place, the second pessary preventing the expulsion either of the pessary or of the hemorrhoid, the muscles apparently clasping the two pessaries around and between them, holding them in place and preventing the enlarged veins from falling. In case of obstinate constipation, the corrugated pessary is used. When the parts are inflamed, the use of cold water in the hollow pessaries affords relief. When the rectum has become relaxed, the use of some astringent ointment, as Ung. Gallæ Co., in the hollow-perforated pessary, is indicated. The pessaries are to be removed daily, and carefully cleansed.

It is not known to the writer whether originality is claimed by the patient for this ingenious device, but it matters not, if the principle here adopted is as applicable to diseases of the lower end of the rectum as it has been found to be in uterine displacements. Judged from its success in this case, the profession may well utilize these measures in the treatment of an often intractable disorder, and, by modification of the pessary in size and form, afford to the enlarged hemorrhoidal veins, and to the relaxed and often protruding rectal walls, needed pressure and support.

The cost of the pessaries is trifling, and it is apparent that their use will often bring coveted relief. The idea has been explained to several of our prominent physicians, who, while not having any experience in its application to the malady for which the pessaries are intended, endorse the principle upon which they are based. We ask the profession to utilize the hint here given and seek to bestow relief through this simple agency to a class of cases which, heretofore, have tested their ingenuity.

## Figures and Tables

**Fig. 1 f1:**
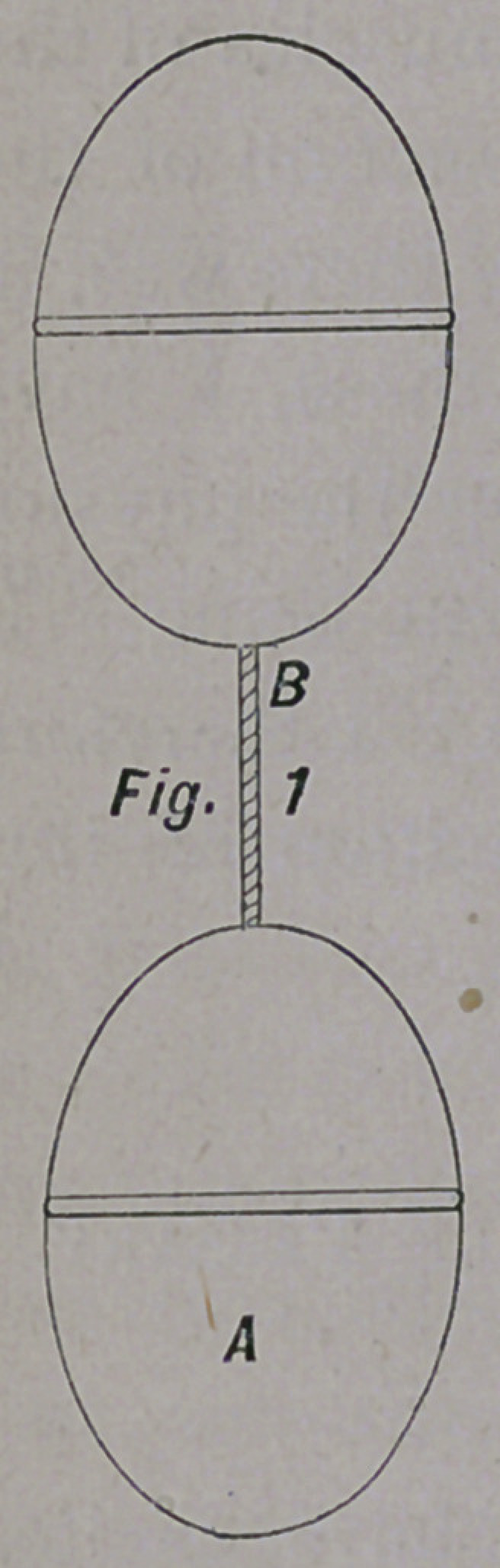


**Fig. 2 f2:**
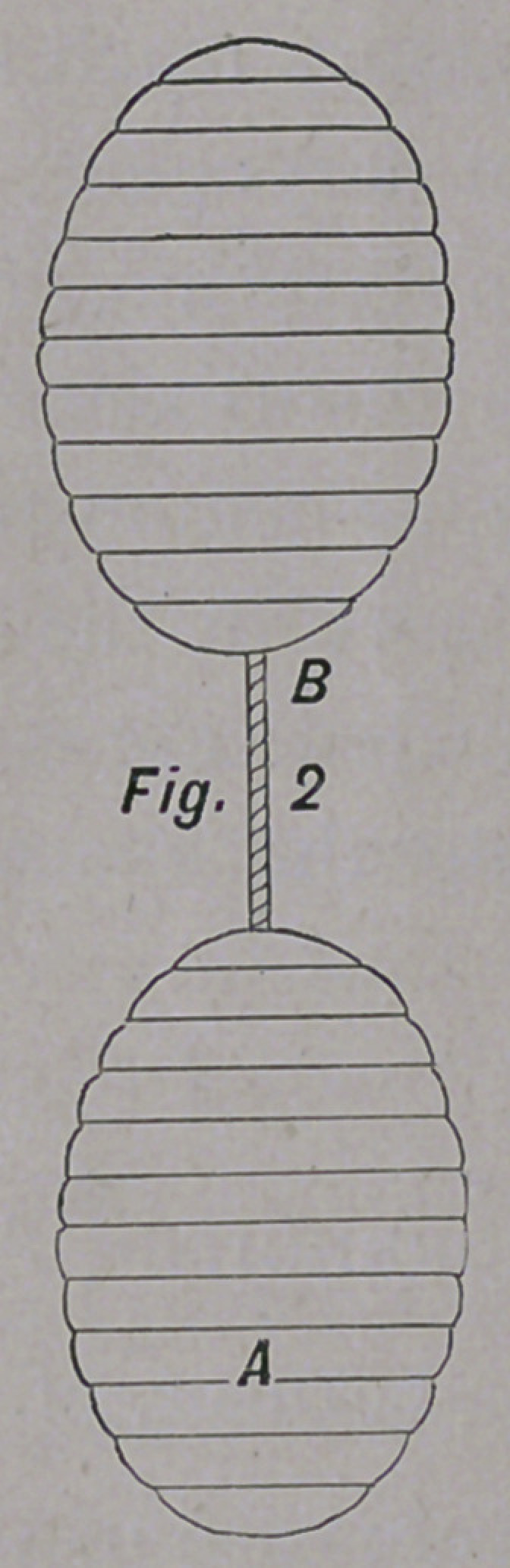


**Fig. 3 f3:**
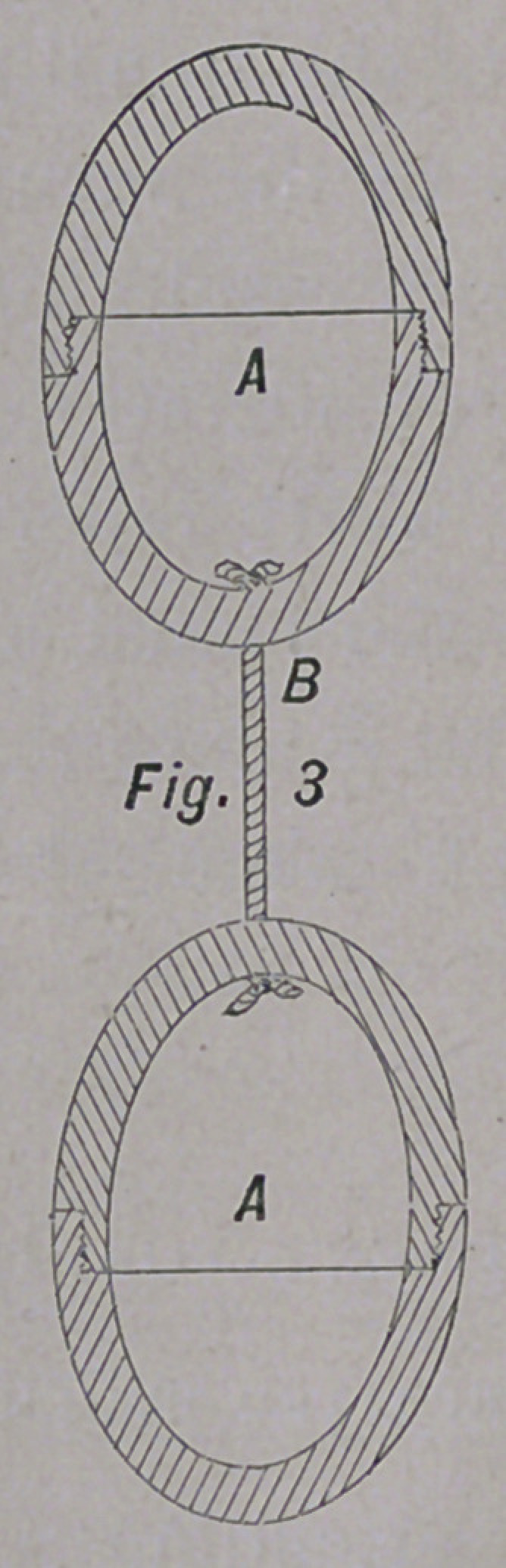


**Fig. 4 f4:**